# Supplementary material for: Composition and Biophysical Properties of the Sorting Platform Pods in the Shigella Type III Secretion System
Source: Front Cell Infect Microbiol. 2021 Jun 3;11:682635. doi: 10.3389/fcimb.2021.682635 (PMC8211105; doi:10.3389/fcimb.2021.682635)
Supplement: Supplementary file 1 [file DataSheet_1.pdf]

**Supplementary Material**

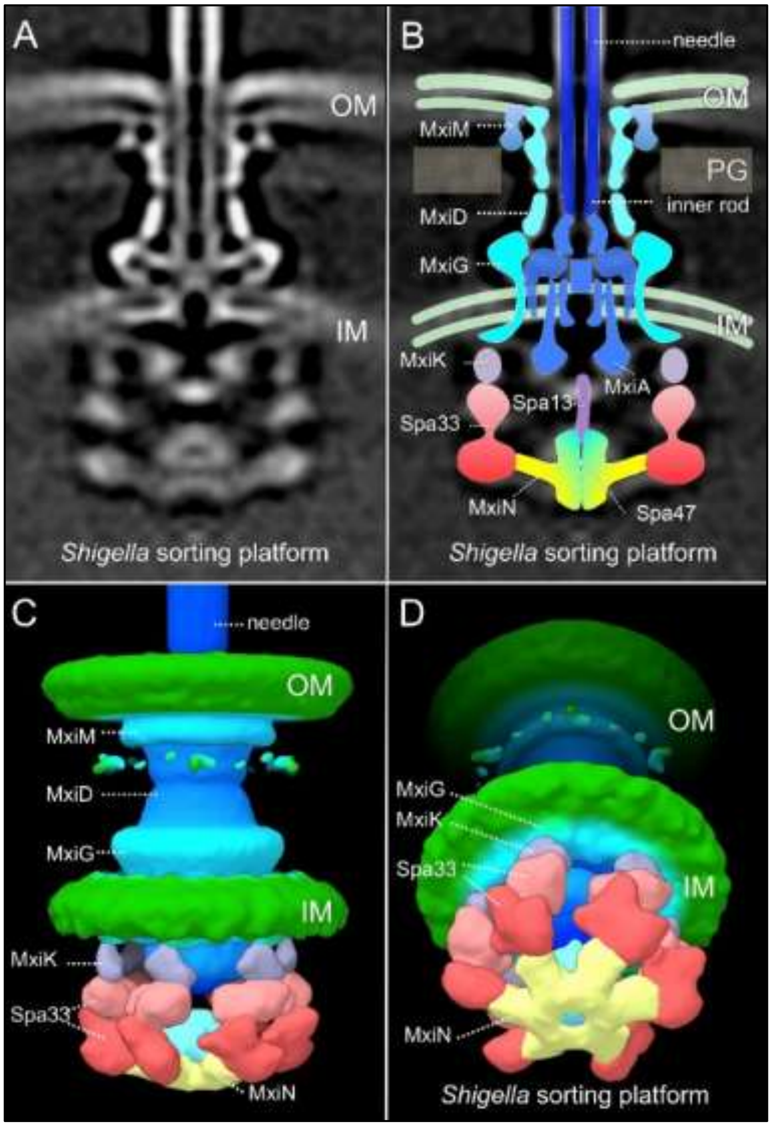


**Supplemental Figure S1. Arrangement of the SP "pods".** A cryo-ET image of the *in situ* wild-type *Shigella* injectisome is shown in **Panel A**. **Panel B** shows the position of the major injectisome components relative to the inner (IM) and outer (OM) membranes and peptidoglycan cell wall (PG) indicated. Based on the cryo-ET image in **A**, a rendering of the injectisome structure is shown in **Panel C** with MxiK (SctK) being the adaptor protein between the Spa33 (SctQ) pods and the cytoplasmic domain of MxiG (SctD) of the IM ring. **Panel D** shows a tilted version of the injectisome that focuses on the SP with the Spa33 densities (red) making up the bulk of the pods. MxiN (SctL) provides the radial spokes that connect Spa33 to the central hub which is the Spa47 (SctN) ATPase. The Spa33 complex consists of what appears to be two large lobes (**Panels B-D**). This figure was originally presented in Hu B, Morado DR, Margolin W, Rohde JR, Arizmendi O, Picking WL, Picking WD, Liu J (2015) Visualization of the type III secretionsorting platform of *Shigella flexneri*. Proc Natl Acad Sci USA 112:1047-1052 (doi: 10.1073/pnas.1411610112).

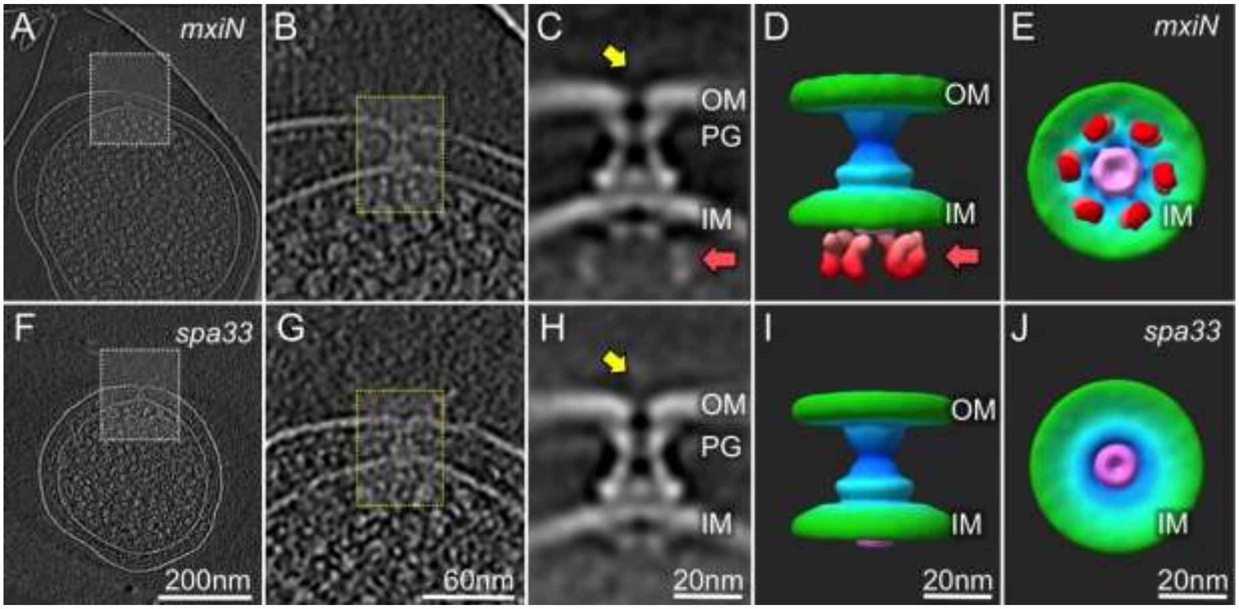


**Supplemental Figure S2. Two injectisome structures from *S. flexneri* minicells lacking MxiN or Spa33**. Representative slices of cryo-ET reconstructions of a Δ*mxiN* minicell **(A)** or a Δ*spa33* minicell **(F)**. The corresponding zoomed-in views are shown in **(B)** and **(G)**, respectively. The averaged structure derived from Δ*mxiN* injectisomes is shown in (**C**), together with two 3-D surface views **(D** and **E)**. The averaged structure derived from Δ*spa33* injectisomes is shown in **(H)**, together with two 3-D surface views **(I** and **J)**. This figure was originally presented in Hu B, Morado DR, Margolin W, Rohde JR, Arizmendi O, Picking WL, Picking WD, Liu J (2015) Visualization of the type III secretionsorting platform of *Shigella flexneri*. Proc Natl Acad Sci USA 112:1047-1052 (doi: 10.1073/pnas.1411610112).

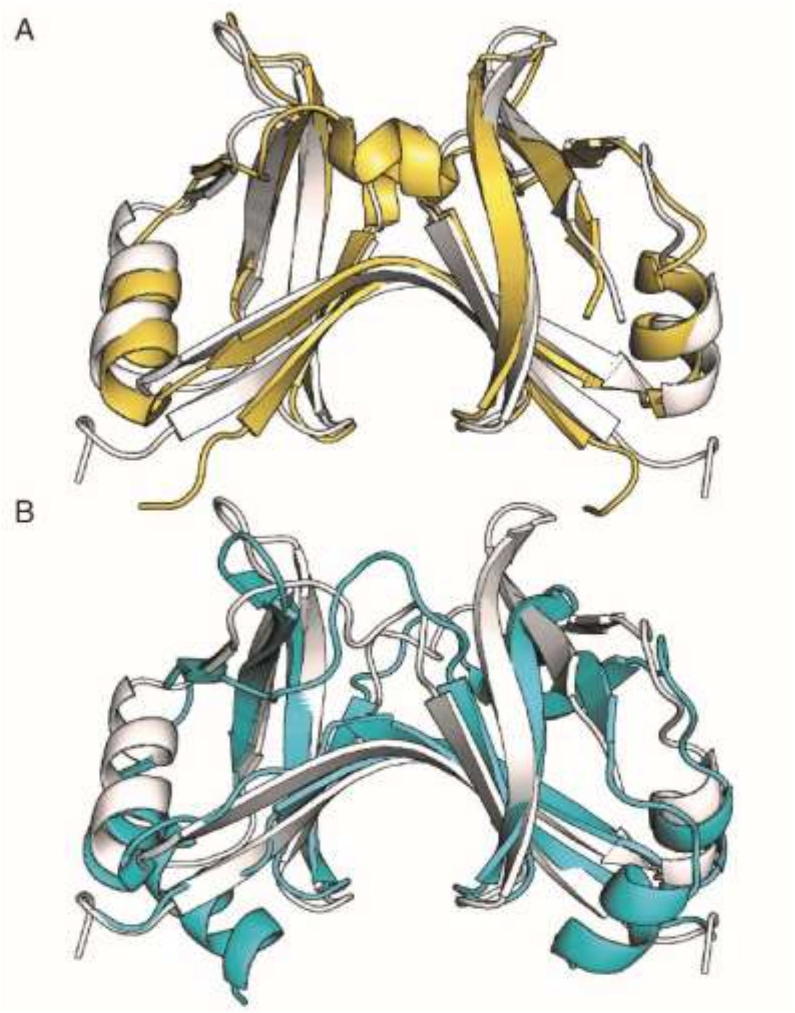


**Supplemental Figure S3. SPOA domain structures. A)** The crystal structures of the injectisome pod protein components Spa33$^{C}$ (PDB: 4TT9) from *Shigella* (white) and SctQ$^{C}$ (PDB: 4YX1) from *Salmonella* (yellow) are shown (RMSD = 1.4 Å). **B)** The Spa33$^{C}$ structure (white) also compares favorably with the flagellar C-ring protein FliN (PDB: 1YAB) from *Thermotoga maritima* (cyan) (RMSD = 2.1 Å).

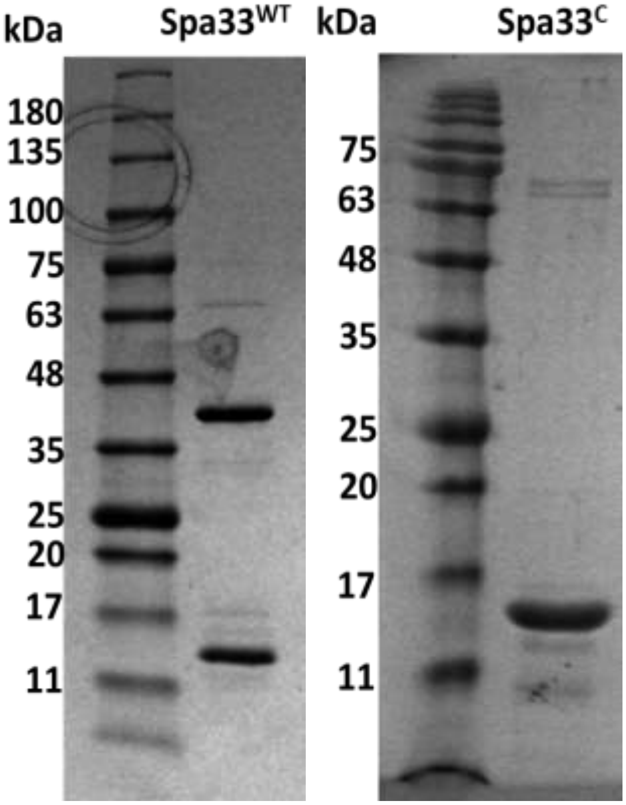


**Supplemental Figure S4. Expression of recombinant Spa33 in *E. coli*.** The final purification products were analyzed by SDS-PAGE. A $His_6$-tagged version of wild-type Spa33 shows two bands on the SDS-PAGE gel (**left**). The bands at approximately 38 kDa and 13 kDa based on the molecular weight markers are $His_6$-tagged $Spa33^{FL}$ and $Spa33^{C}$, respectively. The final purification product of $Spa33^{C}$ is shown in the **right** image with the size being slightly larger than in the **left** due to the presence of a $His_6$ affinity tag. Only one dominant band was visible in this case. Molecular weight markers are shown in the first lane of both Coomassie blue-stained gels.

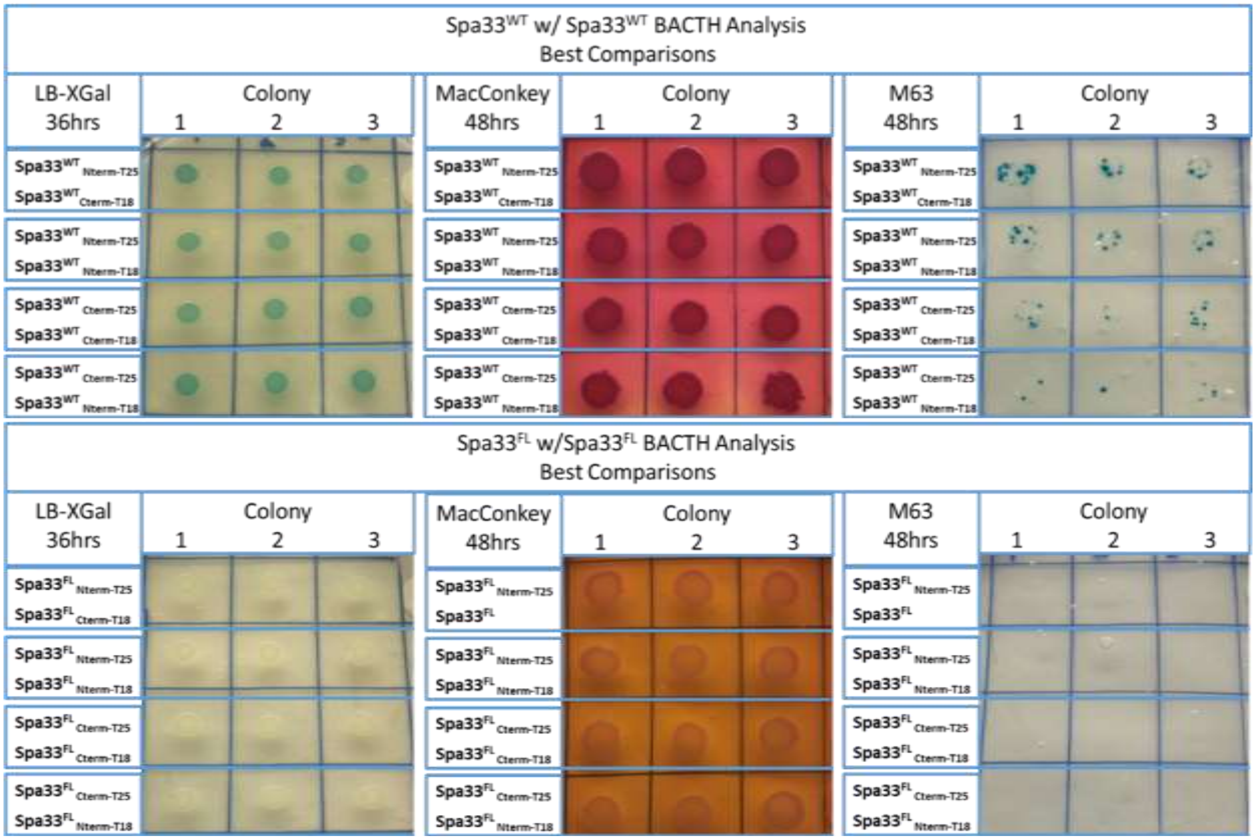


**Supplemental Figure S5. Wild-type Spa33 shows an interaction with wild-type Spa33 while full-length Spa33 (Spa33$^{FL}$) does not show an interaction with another Spa33$^{FL}$ in BACTH analyses.** Interaction among components of wild-type Spa33 was tested using a BACTH analysis (**top**). All combination of wild-type *spa33* with the T18 and T25 vectors were co-transformed into *E. coli* BTH101. All bacterial spots had turned blue on X-Gal plates (**left and right**) and red on MacConkey plate (**middle**). While the entire colony did not turn blue on the M63 plates, punctate blue patches were observed within each overall colony. Then the interactions between Spa33$^{FL}$ and a second copy of Spa33$^{FL}$ was tested (**bottom**). In this case, none of the combinations of Spa33$^{FL}$ fused with T18 or T25 gave the color change that would indicate that they interact.

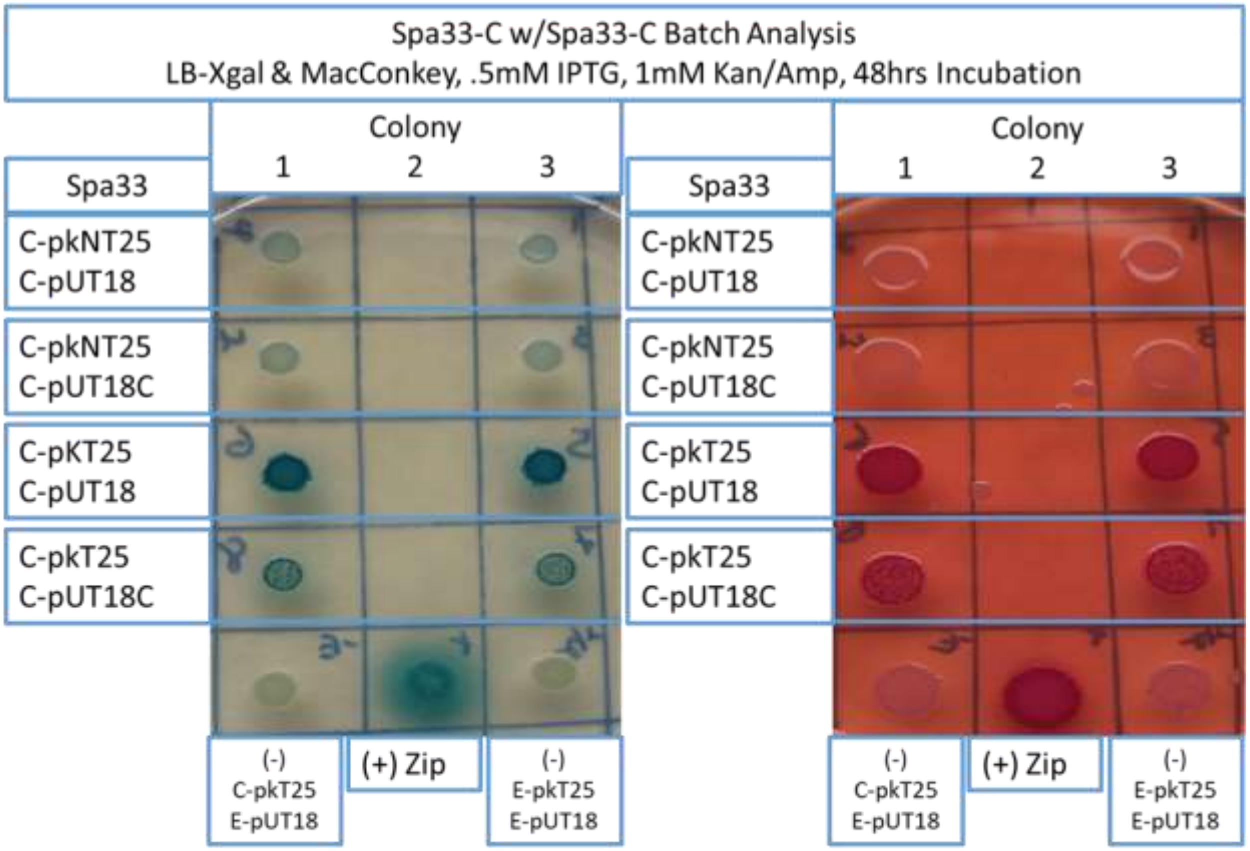


**Supplemental Figure S6. Spa33$^C$ interacts with Spa33$^C$ in BACTH analyses.** Not all combinations of the T18 and T25 vectors with spa33$^C$ used to test for interaction for this dimeric protein were positive. However, *spa33*$^C$ in pKT15 and pUT18 and *spa33*$^C$ in pKT25 and pUT18C clearly showed color changes on the indicator plates. This experiment confirmed that this two-hybrid analysis can detect the formation of Spa33$^C$ dimers.

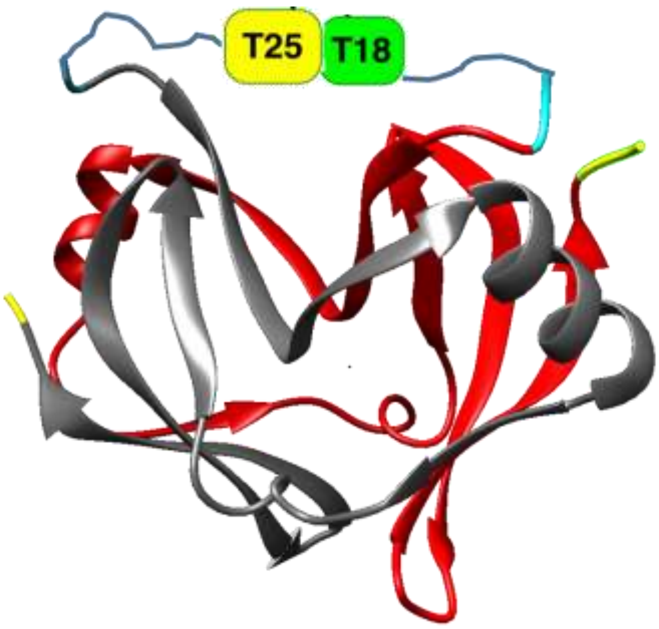


**Supplemental Figure S7. The interacting Spa33$^{C}$ partners must allow for the proper orientation of the CyaA T18 and T25 domains to permit restoration of adenylate cyclase activity.** This figure illustrates how two CyaA subdomains, T18 and T25, in Spa33$^{C}$ may be able to interact and restore CyaA activities in *E. coli* BTH101. T18 and T25 subdomains must be localized in the appropriate location and orientation to restore its enzymatic activity during the interaction. This is why not all combinations in Supplemental Figure S5 restored CyaA β-galactosidase activity. (The Spa33 structure is from PDB ID: 4TT9.)

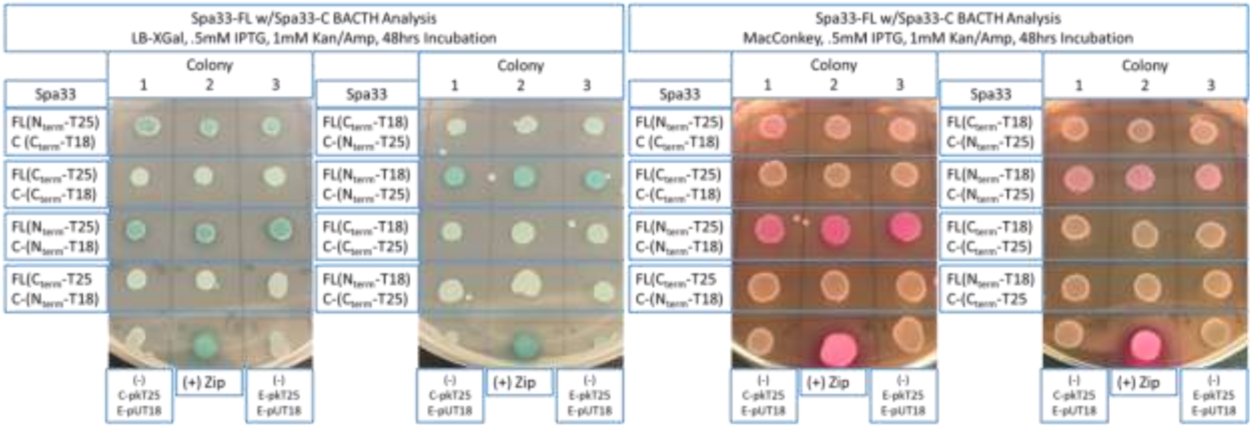


**Supplemental Figure S8. Spa33$^{FL}$ is able to interact with Spa33$^{C}$ in BACTH analyses.** All combinations of *spa33$^{FL}$* and *spa33$^{C}$* in T18 and T25 plasmids were co-transformed into *E. coli* BTH101 strain to test their interactions. Some but not all combinations of these fusion plasmids turned blue and red on LB plate with X-Gal and MacConkey plate with maltose, respectively.

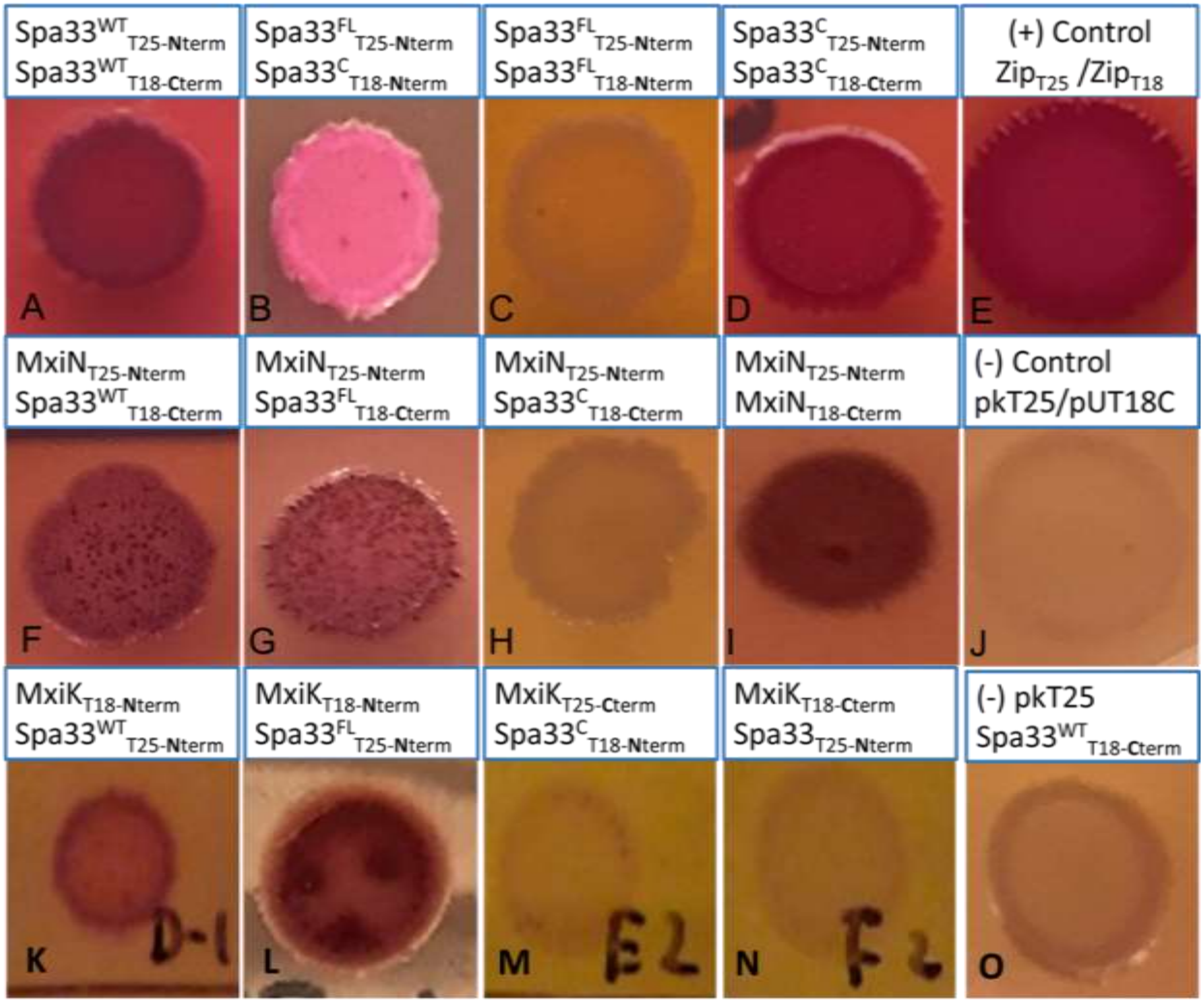


**Supplemental Figure S9. BACTH analysis shows that Spa33 interacts with MxiN and MxiK through Spa33$^{FL}$.** As already shown, wild-type Spa33 can interact with itself, Spa33$^{FL}$ can interact with Spa33$^{C}$, Spa33$^{FL}$ cannot interact with itself and Spa33$^{C}$ can interact with itself (**top row**). In contrast, wild-type Spa33 and Spa33$^{FL}$ interact with MxiN, but Spa33$^{C}$ does not interact with MxiN (and MxiN is able to interact with itself) (**middle row**). Likewise, wild-type Spa33 and Spa33$^{FL}$ interact with MxiK, but Spa33$^{C}$ does not (**bottom row**). Not all BACTH plasmid permutations are shown here, however, in no case was any interaction between Spa33$^{C}$ detected in these analyses.

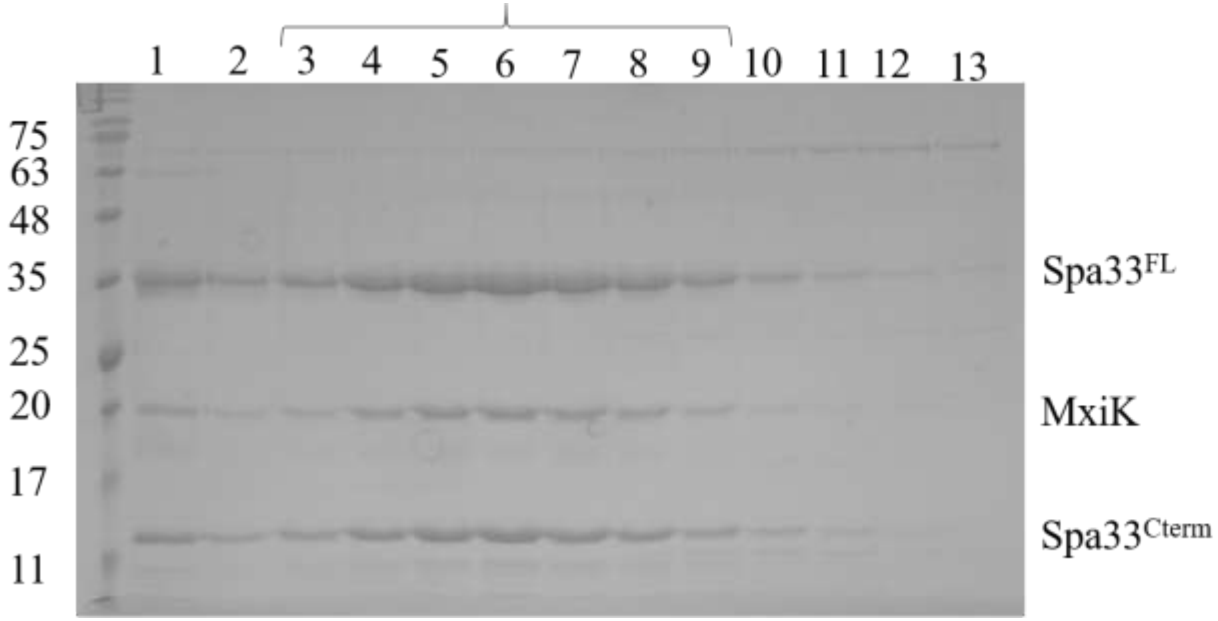


**Supplemental Figure S10. SDS-PAGE analysis of Spa33$^{WT}$-MxiK complex.** Wild-type Spa33 and MxiK were co-expressed in *E. coli* and purified on an IMAC affinity column by virtue of a his-tag at the N-terminus of Spa33. The IMAC fractions of the purified complex was then further purified by size-exclusion chromatography (SEC) on a 200pg column with the eluting fractions shown here. Spa33, which is composed of Spa33$^{FL}$ and Spa33$^{C}$, and MxiK were co-eluted from the purification column. The protein components are indicated at the right and fractions 3 to 9 represent the peak of the co-eluting proteins.

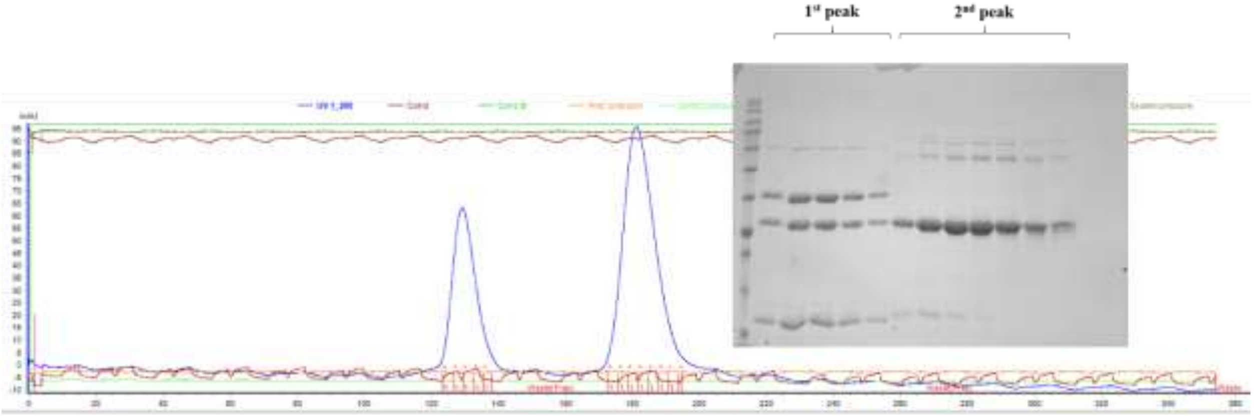


**Supplemental Figure S11. Size-exclusion chromatography profile for the Spa33-MxiN complex (first peak) and residual MxiN (second peak).** MxiN and Spa33 were purified by IMAC with their $His_6$-tags subsequently removed. The two where then mixed with an excess of MxiN with the protein sample then was then subjected to size-exclusion chromatography. The chromatogram showed two different populations were eluted. SDS-PAGE was then used to analyze the eluted proteins. Fractions from the first (higher molecular weight) peak contained Spa33$^{FL}$, Spa33$^{C}$ and MxiN. The second peak exclusively contained the excess MxiN, indicating all of the Spa33 heterotrimer was associated with MxiN.

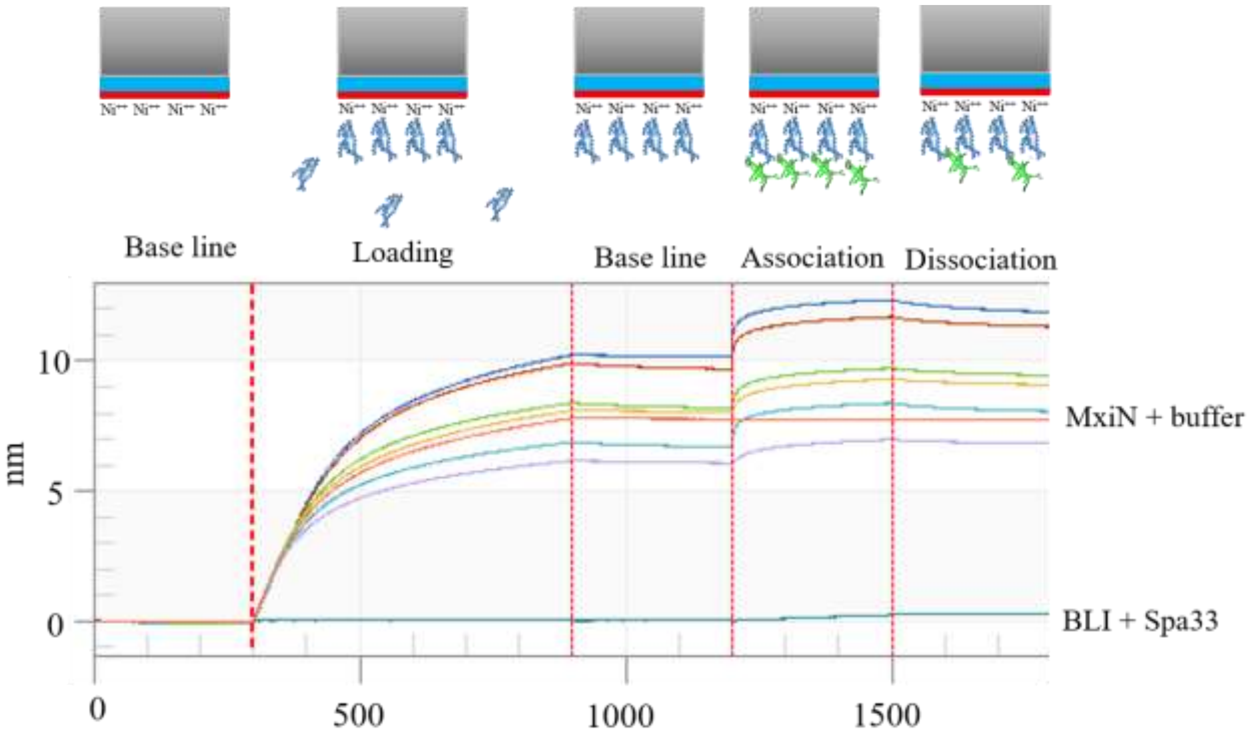


**Supplemental Figure S12. Biolayer Interferometry (BLI) analysis of Spa33 interacting with His$_6$-tagged MxiN.** MxiN with a His$_6$-tag was loaded onto nickel-NTA biosensor tips and a baseline established. Association was then measured in buffer alone and with six different concentrations of wild-type Spa33 (heterotrimer) and the association monitored in real time. The MxiN-containing solution was then replaced with buffer alone and dissociation was then monitored in real time. The bottom baseline trace is the addition of Spa33 to sensor tips without prior loading with His$_6$-tag MxiN.

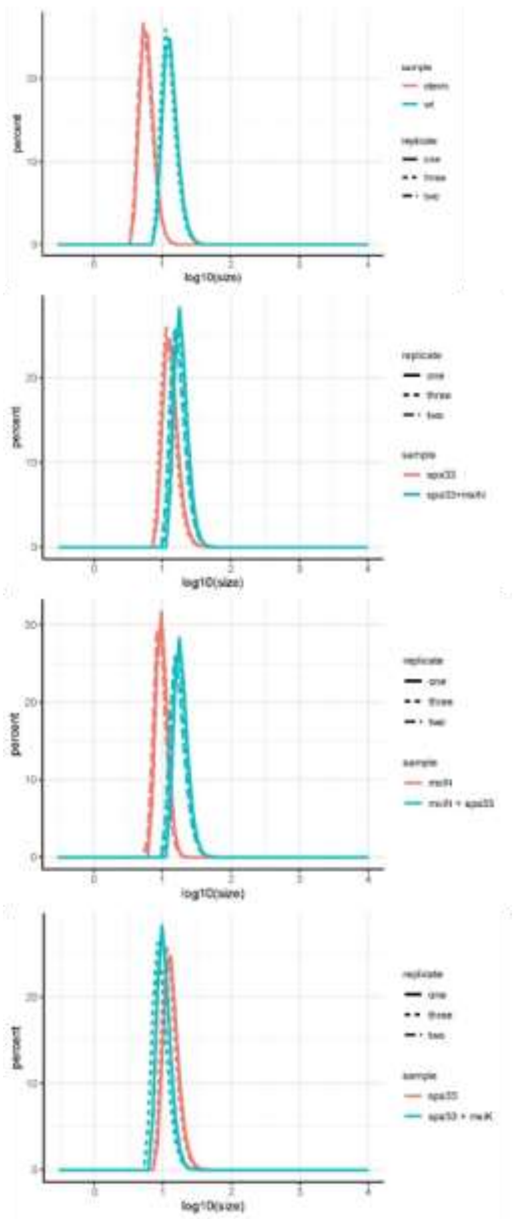


**Supplemental Figure S13. Dynamic light scattering of Spa33-containing complexes.** Raw scans (in triplicate) are shown for wild-type Spa33 versus Spa33$^{C}$ (**top panel**), Spa33 versus the Spa33-MxiN complex (**second panel**), MxiN versus the Spa33-MxiN complex (**third panel**) and Spa33 versus the Spa33-MxiK complex (**bottom panel**).

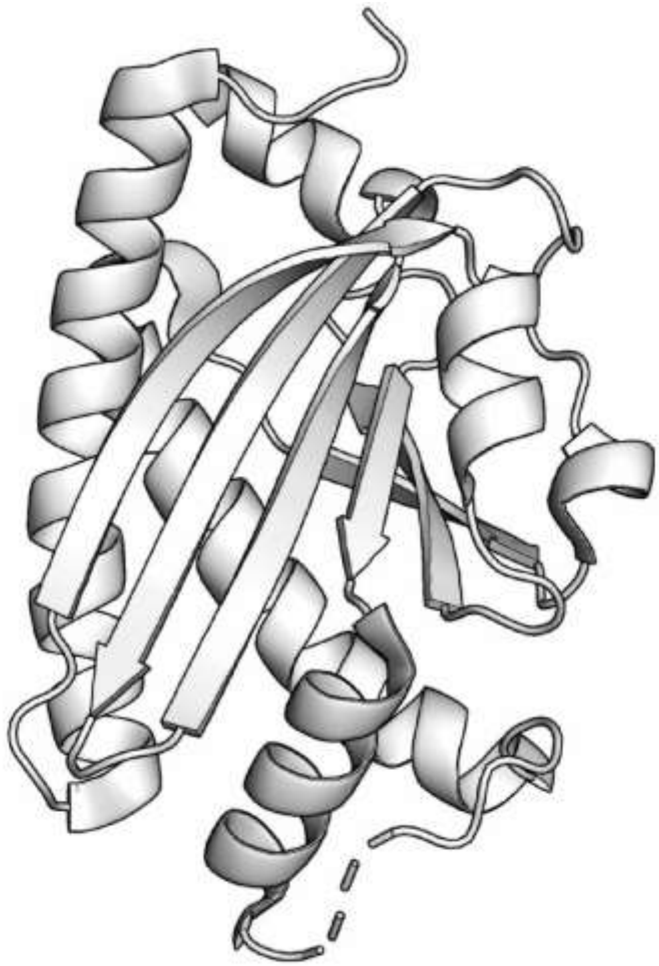

**Supplemental Figure S14. Crystal structure of FliM from *Thermatoga maritima*.** The crystal structure *T. maritima* (PDB: 2HP7) has a greater α-helical content than does FliN and the CD spectrum of the wild-type Spa33 trimer suggests that this is also true for the N-terminal portion of $Spa33^{FL}$.

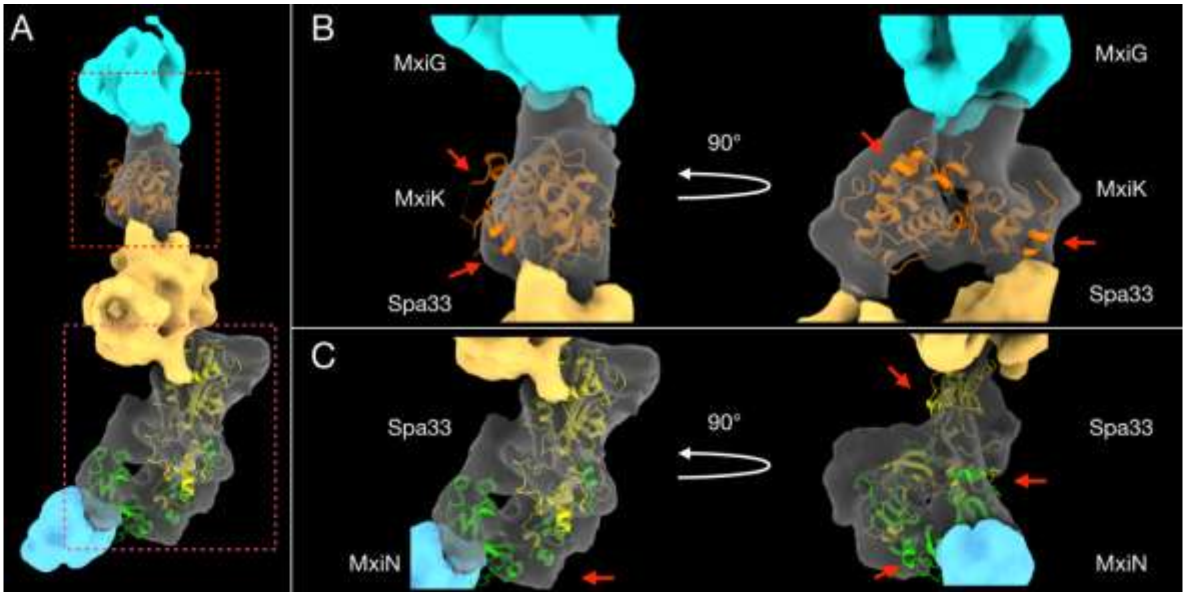


**Supplemental Figure S15. Up close model of an injectisome pod. A)** The 3D surface rendering image of a single pod structure from **Panel B** of **Figure 7** is shown. **B)** The upper red square from **Panel A** is shown up close so that a fit of the crystal structure of *Pseudomonas aeruginosa* SctK can be more easily viewed. The sequence of SctK from *P. aeruginosa* is nearly 20% identical to MxiK and most of its structure (as a monomer) (Muthuramalingam et al. 2020. J. Mol. Biol. 432:166693. doi: 10.1016/j.jmb.2020.10.027) readily fits into the rendering. There are, however, a few areas (indicated by red arrows) that are located outside of the MxiK density. The **right side** is rotated 90˚ . **C)** The lower magenta square from **Panel A** is shown up close in **Panel C**. The model of the Spa33 heterotrimer, based on a model of the FliM/FliN heterotetramer, fits well within the lower of the two main pod densities.
